# Supplementary material for: Motor generated torque drives coupled yawing and orbital rotations of kinesin coated gold nanorods
Source: Commun Biol. 2022 Dec 20;5:1368. doi: 10.1038/s42003-022-04304-w (PMC9767927; doi:10.1038/s42003-022-04304-w)
Supplement: Supplementary file 2 — Supplementary Information [file 42003_2022_4304_MOESM2_ESM.pdf]

Supplementary Information for

Motor generated torque drives coupled yawing and orbital rotations of kinesin coated gold nanorods

Mitsuhiro Sugawa*, Yohei Maruyama, Masahiko Yamagishi, Robert A. Cross and Junichiro Yajima*

Corresponding to Mitsuhiro Sugawa, Junichiro Yajima
Email: mitsuhiro.sugawa@bio.c.u-tokyo.ac.jp, yajima@bio.c.u-tokyo.ac.jp

This PDF file includes:

Supplementary Note 1
Supplementary Figures 1 to 7

Supplementary Note 1: Pseudo code

Pseudo code of Monte Carlo simulation performed in the main text.

Parameters

Asymmetric factor: α_x, α_y
Time constants of the on and off states: $\tau_{\text{on}}, \tau_{\text{off}}$
Standard deviation of Gaussian: σ
Torque generated by one particle: T_{unit}
Total torque: T_{total}
Arrangement of joints on cargo at initial time: (x_i^{j0}, y_i^{j0}) $(i = 1 \ldots N)$
Simulation time: *duration*

Particle states (position, on/off state, dwell time count)

Position: (x_i^p, y_i^p)
On/off state: $state_i^p = \text{"on"}\ or\ \text{"off"}$
Dwell time count: $dwell_i^p$

Cargo state (position, orientation)

Position: (x^c, y^c)
Orientation (degree): θ^c

Binding sites (position)

Position: $(\text{integer}, \text{integer})$

Main function

SET parameters
SET initial states of particle states $(x_i^p, y_i^p, state_i^p, dwell_i^p)$ and cargo state(x^c, y^c, θ^c)
WHILE *t* ← 0 to *duration*
    DO "**Calculate force and moment balance**"
    DO "**Move particles in the off state**"
    DO "**Calculate next particle states**"
    IF all particles are in the off state. **THEN**
        END WHILE
    END IF
    t = t + 1
END WHILE
RETURN particle state sequence $\{(x_i^p, y_i^p, state_i^p, dwell_i^p \mid i = 1 \ldots N)_{time} | time = 0 \ldots t\}$
and cargo state sequence $\{(x^c, y^c, \theta^c)_{time} | \quad time = 0 \ldots t\}$

<u>Subfunction “Calculate force and moment balance”</u>

INPUT particle states $\left(x_i^p, y_i^p, state_i^p, dwell_i^p\right)$, arrangement of joints $\left(x_i^{j0}, y_i^{j0}\right)$, cargo state (x^c, y^c, θ^c), and total torque (T_{total})

WHILE $|\sum_i m_i(\theta^c) - T_{\text{total}}| < 0.5$

  DO calculate present joint positions (x_i^j, y_i^j) from arrangement of joints (x_i^{j0}, y_i^{j0}) and cargo state (x^c, y^c, θ^c)

  DO calculate force-balanced position by

$$(x^c,\ y^c)\ += \left(\frac{\sum_i (x_i^p - x_i^j)}{N_{\text{on}}}, \frac{\sum_i (y_i^p - y_i^j)}{N_{\text{on}}} \right),\ \{x_i^p, y_i^p, x_i^j, y_i^j \mid\ state_i^p = \text{"on"}\}$$

  DO calculate present joint positions (x_i^j, y_i^j) from arrangement of joints (x_i^{j0}, y_i^{j0}) and cargo state (x^c, y^c, θ^c)

  DO calculate moment by

$$m_i = (x_i^j - \frac{\sum_i x_i^j}{N_{\text{on}}},\ y_i^j - \frac{\sum_i y_i^j}{N_{\text{on}}})\ \times (x_i^p - x_i^j,\ y_i^p - y_i^j)$$

  IF $|\sum_i m_i(\theta^c) - T_{\text{total}}| < 0.5$ **THEN END WHILE**

  ELSE $\sum_i m_i(\theta^c) - T_{\text{total}} < 0$ **THEN** $\theta^c -= 0.05$

  ELSE $\sum_i m_i(\theta^c) - T_{\text{total}} > 0$ **THEN** $\theta^c += 0.05$

END WHILE

RETURN cargo state (x^c, y^c, θ^c)

<u>Subfunction “Move particles in the off state”</u>

INPUT particle states $(x_i^p, y_i^p, state_i^p, dwell_i^p)$, arrangement of joints (x_i^{j0}, y_i^{j0}) and cargo state (x^c, y^c, θ^c)

DO calculate present joint positions (x_i^j, y_i^j) from joint arrangement (x_i^{j0}, y_i^{j0}) and cargo state (x^c, y^c, θ^c)

FOR $i \leftarrow 1$ to N

  IF $state_i^p = \text{"off"}$ **THEN**

    DO generate random values $(x_{\text{rnorm}}, y_{\text{rnorm}})$ from Gaussian with center position $\left(x_i^j, y_i^j\right)$ and standard deviation σ

    $\left(x_i^p, y_i^p\right) = (x_{\text{rnorm}}, y_{\text{rnorm}})$

  END IF

END FOR

RETURN particle states $(x_i^p, y_i^p, state_i^p, dwell_i^p)$

Subfunction "Calculate next particle states"

1. **INPUT** particle states $\left(x_i^p, y_i^p, state_i^p, dwell_i^p\right)$, torque generated by one particle (T_{unit}) and total torque (T_{total})
2. **Initialize** number of particles taking a step $N_{\mathrm{step}} = 0$ and total torque $T_{\mathrm{total}} = 0$
3. **FOR** $i \leftarrow 1$ to N
4. $dwell_i^p \ -= 1$
5. **IF** $dwell_i^p = 0$ **THEN**
6. **Invert** $state_i^p$
7. **IF** $state_i^p = \text{"on"}$ **THEN**
8. $dwell_i^p = integer(\text{random value from exponential with parameter } \tau_{\mathrm{on}})$
9. **DO** "**Calculate new binding site**"
10. $N_{\mathrm{step}} += 1$
11. **ELSE** $state_i^p = \text{"off"}$ **THEN**
12. $dwell_i^p = integer(\text{random value from exponential with parameter } \tau_{\mathrm{off}})$
13. **DO** generate random values $(x_{\mathrm{rnorm}}, y_{\mathrm{rnorm}})$ from Gaussian with center position $\left(x_i^p, y_i^p\right)$ and standard deviation σ
14. $\left(x_i^p, y_i^p\right) = (x_{\mathrm{rand}}, y_{\mathrm{rand}})$
15. **END IF**
16. **END IF**
17. **END FOR**
18. $T_{\mathrm{total}} = T_{\mathrm{unit}} \times N_{\mathrm{step}}$
19. **RETURN** particle states $(x_i^p, y_i^p, state_i^p, dwell_i^p)$ and total torque (T_{total})

Subfunction "Calculate new binding site"

1. **INPUT** particle state $(x_i^p, y_i^p, state_i^p, dwell_i^p)$
2. **IF** $x_i^p \bmod (1) > \alpha_x$ **THEN** $x_i^p = integer\left(x_i^p\right) + 1$
3. **ELSE THEN** $x_i^p = integer\left(x_i^p\right)$
4. **END IF**
5. **IF** $y_i^p \bmod (1) > \alpha_y$ **THEN** $y_i^p = integer\left(y_i^p\right) + 1$
6. **ELSE THEN** $y_i^p = integer\left(y_i^p\right)$
7. **END IF**
8. **RETURN** particle state $(x_i^p, y_i^p, state_i^p, dwell_i^p)$

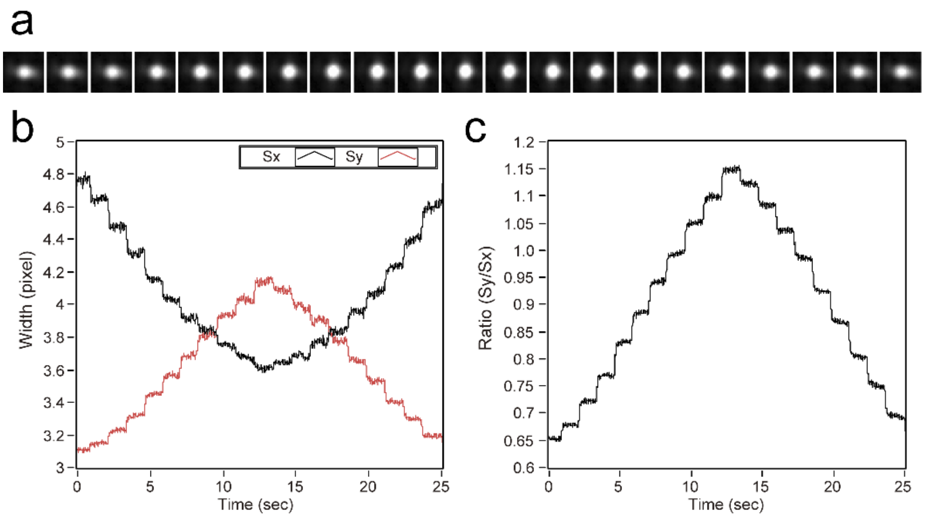

Supplementary Figure 1. Calibration of GNR displacements along the *Z*-axis.

a Scattered images of a single GNR bound to the coverslip, taking 50-nm steps along the Z-axis. **b** Time trajectories of the widths of the GNR spot images in **a** along the *X*-axis (S_X, black line) and *Y*-axis (S_Y, red line). The widths were the standard deviations obtained by 2D Gaussian fits. **c** Time trajectory of the ratio of S_Y to S_X, which represents the *Z*-displacement of the GNR.

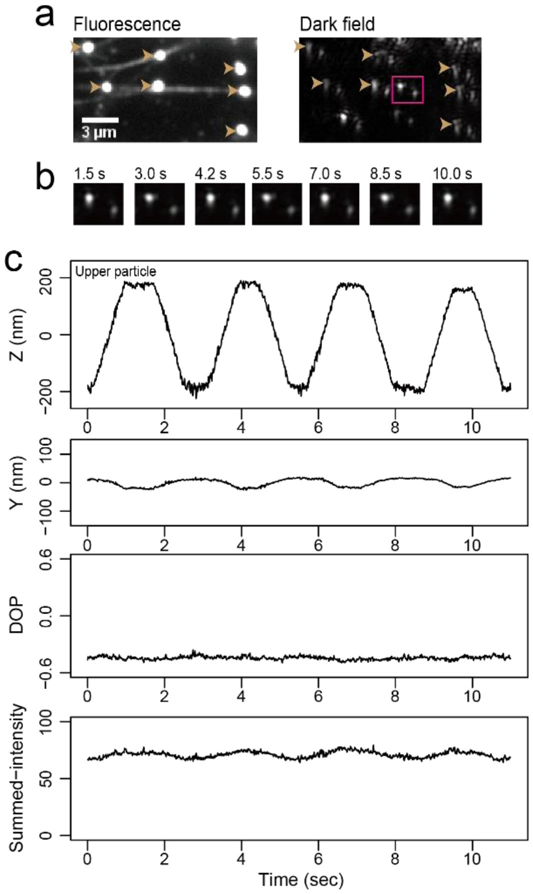

Supplementary Figure 2. Stability of gold-nanorod (GNR) polarization against the *Z*-displacement.

a Fluorescent image of antibody-coated microbeads (arrowheads) and microtubules, and dark-field image of the microbeads (arrowheads) and the GNRs coated with kinesin-1 (Kin1-GNR) (magenta region of interest). Microtubules were suspended on the microbeads via antigen-antibody system for the β tubulin. The single Kin1-coated GNR was bound on the suspended microtubule in the presence of 1 mM AMPPNP. **b** Montage of one pair of the scattered images of the single Kin1-coated GNR shown in the magenta region of interest in **a**. The objective lens repeatedly moved ±400-nm along the *Z*-axis. **c** Time trajectories of the *Z*- and *Y*-displacements, the degree of polarization (DOP), and the summed-intensity of the single Kin1-coated GNR shown in **b**. The *Z*-displacement trajectory was obtained from the upper-left spot in **b**.

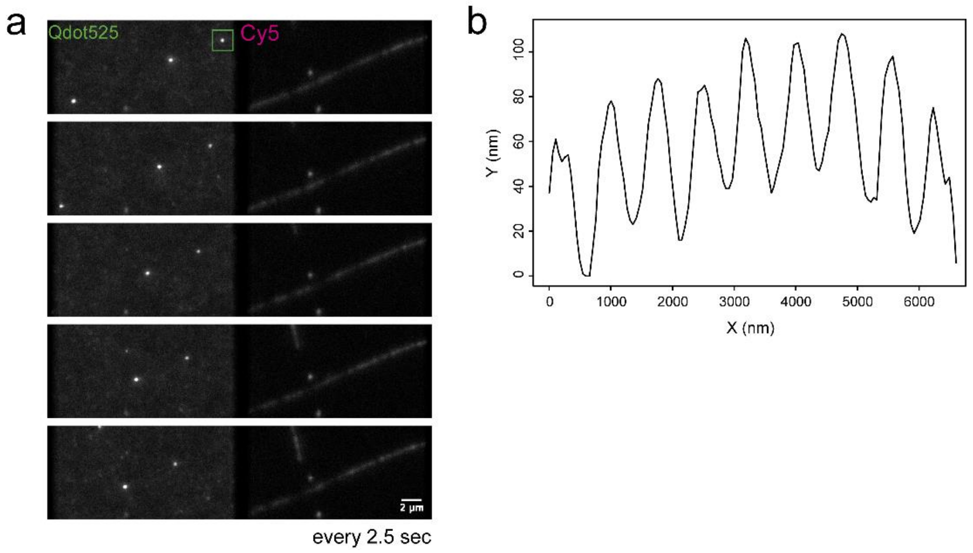

Supplementary Figure 3. Surface-gliding assay of KIF1A.

a Fluorescence images of Qdot525s and a Cy5-labelled microtubule in the surface-gliding assay of KIF1A. **b** Trajectory of the Qdot525 spot in the region of interest in **a**, which exhibits a stable sinewave, suggesting the repetitive corkscrewing motions of the microtubule. A corkscrewing pitch of the microtubule was determined by measuring the *X*-displacement in each period of oscillation along the *Y*-axis. The translational velocity was 0.49 ± 0.02 μm/s (mean ± SD, n = 5 microtubules) and the corkscrewing pitch was 0.89 ± 0.21 μm (mean ± SD, n = 35 revolutions).

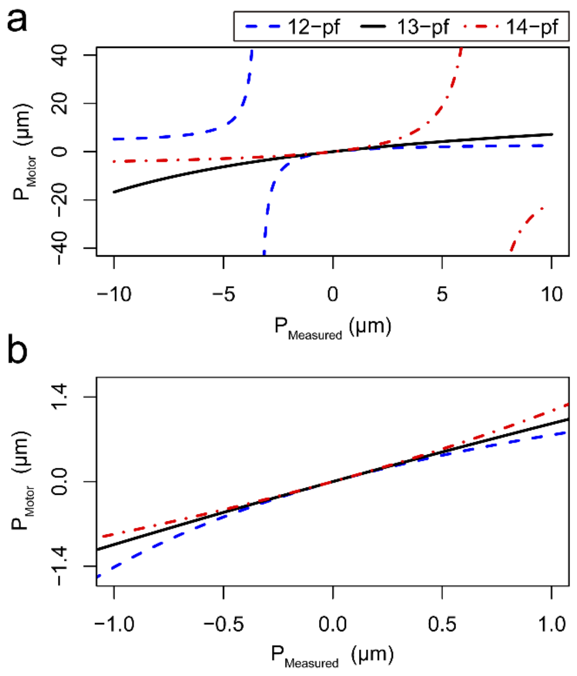

Supplementary Figure 4. Relationship between measured pitches, pitches of motors and supertwist of microtubules.

a, b Pitches of motors (P_{Motor}) as a function of measured pitch ($P_{Measured}$) and supertwists (P_{MT}) of 12-, 13-, and 14-protofilament microtubules (12-pf, 13-pf, and 14-pf, respectively). P_{Motor} is given by $P_{Motor}^{-1} = P_{Measured}^{-1} - P_{MT}^{-1}$. The dashed blue line, the black line, and the doted-and-dashed red line represent the calculation for 12-pf (P_{MT} = −3.4 µm), 13-pf (P_{MT} = −24.8 µm), and 14-pf (P_{MT} = 6.8 µm), respectively.

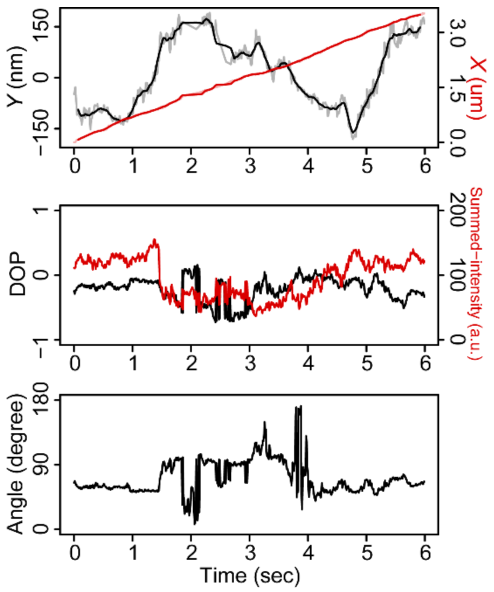

Supplementary Figure 5. Typical time trajectories of kinesin-1 coated GNR.

Time trajectories of the *X*- and *Y*-displacements (upper), the DOP and summed-intensity of the two spots (middle), and the estimated angle (bottom) of the kinesin-1 coated GNR.

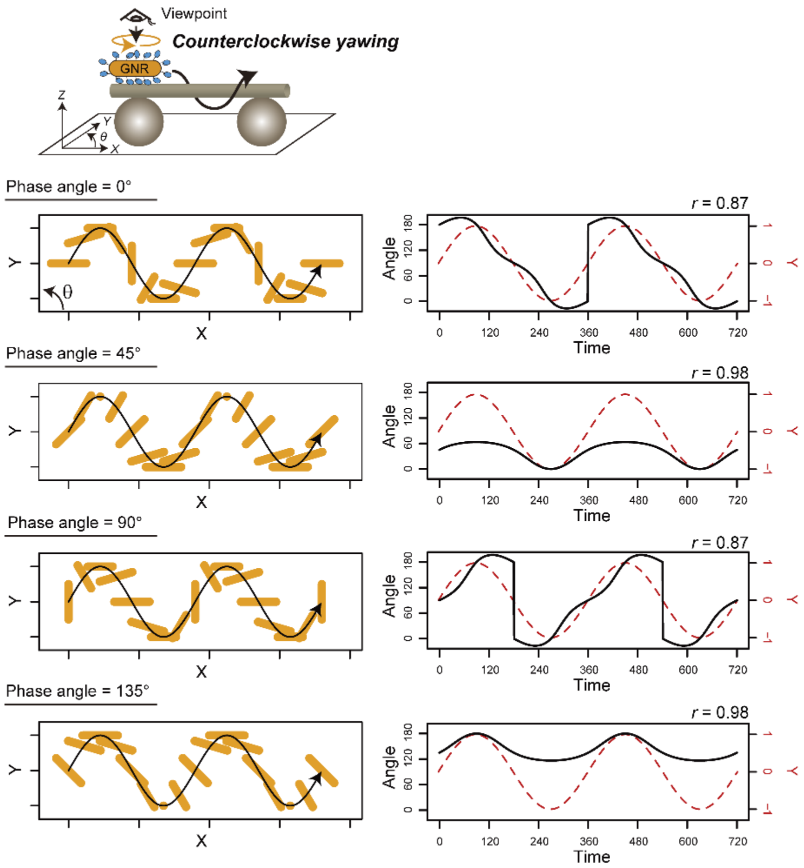

Supplementary Figure 6. Calculated trajectories of a motor-coated GNR in the case of counterclockwise yawing by the proposed model.

Calculation of time trajectories of the angle and the *X*-*Y*-trajectories of a motor-coated GNR using the proposed model with different phase angles (0°, 45°, 90°, and 135°), in which the kinesin-coated GNR rotates 180° counterclockwise about its yaw axis in one period of helical trajectory. See also Supplementary Movie 5.

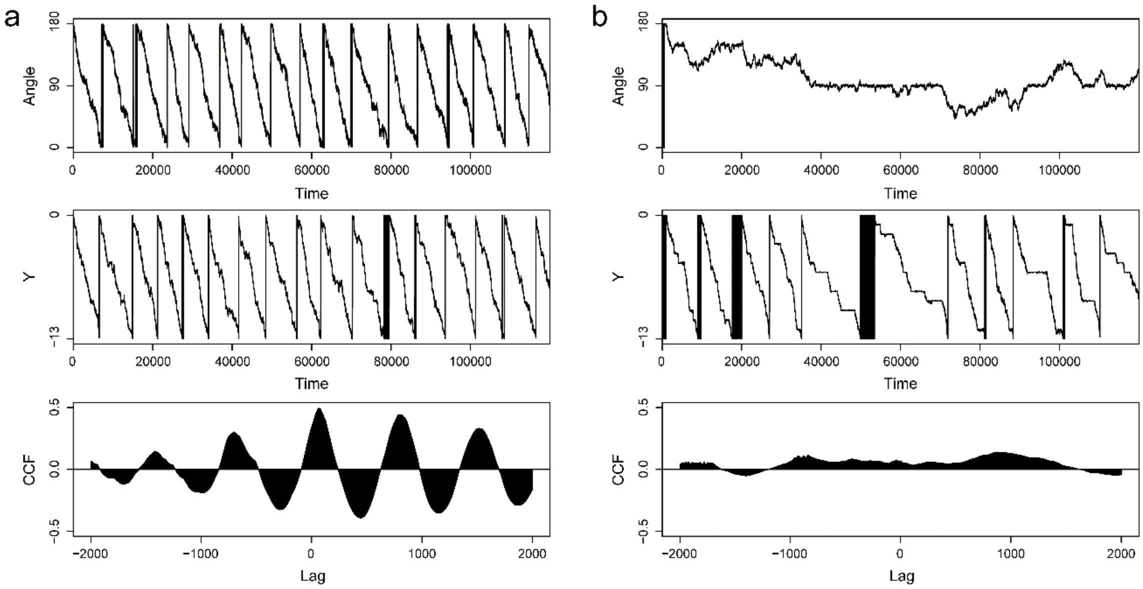

Supplementary Figure 7. Time trajectories of 2D Monte Carlo simulation with and without torque generation.

a, b Time trajectories of the angle and *Y*-displacement, and the cross-correlation function (CCF) between the angle and the Y-displacement of the simulation results shown in Fig. 7f (T_{step} = −24 in **a** and T_{step} = 0 in **b**). The axes of the angle and the *Y*-direction are converted to 180°- and 13-lattice periods, respectively.
