# Supplementary material for: Motor generated torque drives coupled yawing and orbital rotations of kinesin coated gold nanorods
Source: Commun Biol. 2022 Dec 20;5:1368. doi: 10.1038/s42003-022-04304-w (PMC9767927; doi:10.1038/s42003-022-04304-w)
Supplement: Supplementary file 3 — Description of Additional Supplementary Files [file 42003_2022_4304_MOESM3_ESM.pdf]

Description of Additional Supplementary Files

File name: Supplementary Data 1
Description: Data used to generate the charts and graphs in the main figures.

File name: Supplementary Movie 1
Description: The KIF1A-coated GNR moving around the suspended microtubule. The movie shows scattered-light images of one KIF1A-coated GNR around the microtubule in the presence of 2 mM ATP. 10 ms intervals, ×1 speed. The image is 3.8 µm high and 7.5 µm wide.

File name: Supplementary Movie 2
Description: Non-rotation of the GNR about its yaw axis during the left-handed helical motion. The movies show that the GNR does not rotate about its yaw axis during the lefthanded helical motions, with different initial phase angles (0, 45, 90 and 135 degree) of the model.

File name: Supplementary Movie 3
Description: GNR unidirectionally rotates about its yaw and roll axes during the shortpitch helical motion around the filament long axis.

File name: Supplementary Movie 4
Description: Clockwise rotation of the GNR about its yaw axis during the left-handed helical motion. The movies show that the GNR unidirectionally rotates CW about its yaw axis during the left-handed helical motions, with different initial phase angles (0, 45, 90 and 135 degree) of the model.

File name: Supplementary Movie 5
Description: Counterclockwise rotation of the GNR about its yaw axis during the left-handed helical motion. The movies show that the GNR unidirectionally rotates CCW about its yaw axis during the left-handed helical motions, with different initial phase angles (0, 45, 90 and 135 degree) of the model.

File name: Supplementary Movie 6
Description: An unidirectional rotation of the short microtubule The movie shows that the short microtubule unidirectionally rotates on ZEN4-coated GNR while only its end is in contact with the GNR. 100 ms intervals, ×5 speed. The image is 6.4 µm high and 9.4 µm wide.
